# Evolution of Physicochemical Parameters during the Thermal-Based Production of *Água-mel*, a Traditional Portuguese Honey-Related Food Product

**DOI:** 10.3390/molecules27010057

**Published:** 2021-12-23

**Authors:** Teresa Cavaco, Ana Cristina Figueira, Raúl González-Domínguez, Ana Sayago, Ángeles Fernández-Recamales

**Affiliations:** 1 AgriFood Laboratory, Faculty of Experimental Sciences, University of Huelva, 21007 Huelva, Spain; tmgcavaco@gmail.com (T.C.); raul.gonzalez@dqcm.uhu.es (R.G.-D.); ana.sayago@dqcm.uhu.es (A.S.); 2 International Campus of Excellence CeiA3, University of Huelva, 21007 Huelva, Spain; 3 Department of Food Engineering, Institute of Engineering, University of the Algarve, Campus da Penha, 8005-139 Faro, Portugal; afiguei@ualg.pt; 4 Research Centre for Tourism, Sustainability and Well-Being (CinTurs), University of Algarve, Campus of Gambelas, 8005-139 Faro, Portugal

**Keywords:** *água-mel*, thermal processing, 5-hydroxymethylfurfural, sugars, color, kinetics

## Abstract

The purpose of this work was to investigate the physicochemical changes occurring during the thermal-based production of *água-mel*, a traditional Portuguese honey-related food product. The refractive index, color parameters (hue angle, H°; chroma, C*), and the content of total reducing sugars, glucose, fructose, total brown pigments, and 5-hydroxymethylfurfural were monitored along the entire production process, and their evolution was kinetically modelled. Thermal processing caused a gradual decrease in sugars, which was accompanied by the formation of brown pigments and 5-hydroxymethylfurfural, increased concentration of soluble solids as evaluated through refractive index measurements, as well as the appearance of darker colors. In particular, a zero-order kinetic model could explain the changes in H° and reducing sugars, while the evolution of refractive index, brown pigments, 5-hydroxymethylfurfural, C*, fructose, and glucose were best fitted using a first-order kinetics model.

## 1. Introduction

*Água-mel* is a honey-based foodstuff that has been produced since ancient times in south Portugal [1,2,3,4], although a similar product, *abbamele*, is also produced in Sardinia (Italy) [5,6]. Both Mediterranean honey-like groceries result from the valorization of by-products from the honey industry. After honey is extracted, honeycombs are crumbled and scalded with water. The resulting solution, to which orange or lemon rind are usually added as flavorings, is heated at 100 to 108 °C until an appropriate concentration of soluble solids is attained (70–77° Brix) [1,2,3,4,5,6]. However, it should be noted that this production process does not follow any pre-established methodology, being extremely subjective and dependent on the producer´s tradition [1]. This, together with the complex composition of honey, makes the implementation of reliable quality-control procedures necessary to assess the physicochemical properties of the final product and the changes occurring during food processing.

The initial honey solution mainly consists of sugars, predominantly glucose and fructose, and low proportions of other inorganic and organic materials, namely proteins, amino acids, polysaccharides, organic acids, different minerals, as well as pollen and wax [7]. Thermal processing during *água-mel* production results in changes in numerous physicochemical parameters, including decreased water activity and moisture content, increased electrical conductivity and free acidity, and decreased pH [6]. In particular, it has been reported that the moisture content varies substantially, and mainly depends on the water content of raw materials (i.e., honey and honeycombs), the amount of water added during processing, and the temperature and length of the heating process [6]. Heating, unfortunately, also affects the levels of thermolabile components and the formation of Maillard reaction products, which may have a detrimental effect on the quality of the final product [8,9]. The Maillard reaction is a non-enzymatic browning process, which consists of a complex network of reactions involving carbonyl and amino compounds, such as reducing sugars and amino acids. This reaction is responsible for the formation of colorants and flavor compounds during the food heating process [6,10]. In this respect, it has been reported that *água-mel* normally contains high levels of 5-hydroxymethylfurfural (HMF) and other non-enzymic browning reaction products, whereas the content of amino acids and invertase activities dramatically decrease due to the thermal treatment [1,5,6].

In this context, the main objective of this work was to describe the physicochemical changes occurring during the thermal-based production of *água-mel*, a traditional Portuguese product, in terms of sugars, brown pigments, color parameters, and soluble solids.

## 2. Materials and Methods

### 2.1. Água-mel Sampling

*Água-mel* samples were obtained from a local traditional producer located in the Algarve region (south Portugal), as previously described by Figueira and Cavaco [1]. During the entire thermal processing lasting for 400 min at 100 °C, three *água-mel* aliquots were taken at 15 min intervals until the final product was obtained. Samples were placed in airtight glass jars and kept in the dark at room temperature until further analysis. Prior to any analytical determination, the samples were homogenized for 5 min using an Ultra-Turrax^®^ mixer (IKA–Labortechnik, Staufen, Germany). All the determinations were performed in triplicate.

### 2.2. Physicochemical Analyses

#### 2.2.1. Refractive Index

The refractive index (RI) of *água-mel* samples was measured using an Atago NAR-1T refractometer (Atago^TM^, Bellevue, WA, USA) equipped with a direct reading display. All the measurements were performed at room temperature, and the readings were corrected for a standard temperature of 20 °C by considering a correction factor of 0.00023 °C^−1^, as previously described for honey [11].

#### 2.2.2. Reducing Sugars

The total reducing sugar (RS) content was determined by applying the 3,5-dinitrosalicylic acid (DNS) method according to Saxena et al. [12]. The method is based on the reduction of DNS by the reducing sugars present in the sample to generate 3-amino-5-nitrosalicylic acid, which results in the formation of a reddish–orange coloration which is measured spectrophotometrically at 540 nm. To this end, one milliliter of *água-mel* dissolved in water (1 mg mL^−1^) was mixed with an equal volume of DNS reagent and incubated for 10 min in a boiling water bath. The mixture was allowed to cool to room temperature and then mixed with 7.5 mL of Milli Q water (Millipore, Billerica, MA, USA). Finally, the absorbance was measured at 540 nm using an UV/Vis spectrophotometer (Hitachi U-2000, Tokyo, Japan). Standard glucose solutions, within the concentration range 0–1 mg mL^−1^, were used to obtain a calibration curve. The results were expressed as milligrams of glucose equivalents per milliliter of *água-mel*.

#### 2.2.3. Determination of Individual Sugars

Samples (0.5 g) were homogenized with 10 mL of an acetonitrile:water mixture (80:20, *v*/*v*) at room temperature, centrifuged at 5337× *g* for 20 min at 21 °C, and finally filtered through a 0.45 μm filter [13]. Then, the individual sugars (i.e., fructose, glucose and sucrose) were analyzed by high-performance liquid chromatography (HPLC) by direct injection of 20 μL of the filtered solution, according to the method described by Orian [14]. For this purpose, we used a Jasco LG-1580-04 chromatograph equipped with a solvent pump model PU-2080 (Jasco, Easton, MD, USA), and a refractive index detector (Knauer, Berlin, Germany). The separation was performed by using a NH_2_-bonded column (4.6 mm × 250 mm, 5 μm) for carbohydrate analysis (Merck, Darmstadt, Germany). The mobile phase was acetonitrile:water (80:20, *v*/*v*) delivered at a constant flow rate of 1.0 mL min^−1^. Quantitative determinations were carried out using a standard external calibration method (r^2^ = 0.9710, 0.9950 and 0.9836; for fructose, glucose and sucrose, respectively). Detection (LOD) and quantification (LOQ) limits were calculated based on 3.3 s/slope and 10 s/slope, respectively, where s is the standard deviation on intercept of the calibration curve (fructose: LOD = 0.01 mg mL^−1^, LOQ = 0.18 mg mL^−1^; glucose: LOD = 0.01 mg mL^−1^, LOQ = 0.20 mg mL^−1^; sucrose: LOD = 0.02 mg mL^−1^, LOQ = 0.18 mg mL^−1^).

#### 2.2.4. Color Analyses

The color of each sample was determined by measuring its reflectance spectra using a NeurteK spectro-color tristimulus colorimeter, model LMG 170 (Eibar, Spain). The instrument was standardized, prior to analysis, using a standard tile (plate LZM 268) with reflectance values of X = 84.60, Y = 89.46, Z = 93.85 (white tile); and X = 4.12, Y = 4.38, Z = 4.71 (black tile). The color determination is reported in terms of three parameters: L*, an approximate measurement of lightness, which is the property according to which each color can be considered as an equivalent to a member of the grey scale, between black and white, taking values within the range of 0 to 100, respectively; a*, which takes positive values for reddish colors and negative values for greenish ones; and b*, which takes positive values for yellowish colors and negative values for bluish ones. In turn, these three measured color parameters were converted into C* (chroma) and H° (hue angle) values using the following equations [1,12]:

(1) $$C^{*}={\left[{\left(a^{*}\right)}^{2}+{\left(b^{*}\right)}^{2}\right]}^{1/2}$$

(2) $$H^{{}^{\circ}}=arctan\;(\frac{a^{*}}{b^{*}})$$

H° is represented as an angle ranging from 0° to 360°, so that samples located in the first quadrant (0 to 90°) are red, orange, and yellow; those located in the second quadrant (90 to 180°) are yellow, yellow-green, and green; those located in the third quadrant (180 to 270°) are green, cyan (blue-green), and blue; and return again to red in the fourth quadrant (270 to 360°) [1]. On the other hand, the C* parameter is measured according to the distance from the origin point of coordinates to that of the illuminant (neutral color axis), and it represents the degree of saturation, purity, or intensity of visual color [15].

#### 2.2.5. Determination of Brown Pigments

The formation of brown pigments (BP) was assessed by measuring the absorbance of 4° Brix *água-mel* solutions against water, using a UV-Vis double-beam U-200 spectrophotometer operating at 420 nm (Hitachi, Tokyo, Japan), as described by Turkmen et al. [9].

#### 2.2.6. Quantification of 5-Hydroxymethylfurfural

5-Hydroxymethylfurfural (HMF) was determined by high-performance liquid chromatography with a photodiode array detector, according to the procedure described by Zappala et al. [16]. The analysis was conducted in a Jasco LG-1580-04 liquid chromatograph equipped with a solvent pump model PU-2080, and a photodiode array detector MD-2010/2015 (Jasco, Easton, MD, USA). The *água-mel* samples were filtered through a 0.45 μm filter, and directly injected (20 µL) into a Lichrospher 100 RP-18 column (25 cm × 4 mm, 5 µm) (Merck, Darmstadt, Germany). The mobile phase consisted of methanol:water (10:90, *v*/*v*) delivered at a flow rate of 1 mL min^−1^, and HMF was detected at 285 nm. HMF was quantified based on a standard calibration curve in the range 0.00–0.08 mg L^−1^ (r^2^ = 0.9994). Detection (LOD) and quantification (LOQ) limits were calculated based on 3 s/slope and 10 s/slope, respectively, where s is the standard deviation on intercept of the analytical curve (LOD = 0.05 mg kg^−1^, LOQ = 0.15 mg kg^−1^).

### 2.3. Calculation of Kinetic Parameters

Zero-order and first-order equations were applied to model the kinetics of the physicochemical parameters that were evaluated in this study, in line with previous studies reporting that the evolution of food quality parameters usually follows these behaviors [1,17]. The conventional equations for these kinetic models are expressed by equations 3 and 4, for zero-order kinetics and first-order kinetics, respectively:

P = P_0_ ± K_0_t (3)

P = P_0_ exp ^(±K^_1_^t)^ (4)

where P is the value of the property studied at time t; P_0_ is the value of this property at time zero; k_0_ is the zero-order kinetic constant (in reciprocal minutes); k_1_ is the first-order kinetic constant (in reciprocal minutes); t is the processing time (in minutes); (+) and (−) indicate the formation and degradation of the property, respectively.

### 2.4. Statistical Analysis

The results of the different parameters under study were expressed as mean ± standard deviation (SD) from three determinations. The temporal evolution of the variables under study was analyzed by repeated-measures ANOVA. All statistical analyses were performed using the SPSS software package, version 23.0 (Chicago, IL, USA).

## 3. Results and Discussion

**Figure 1:**
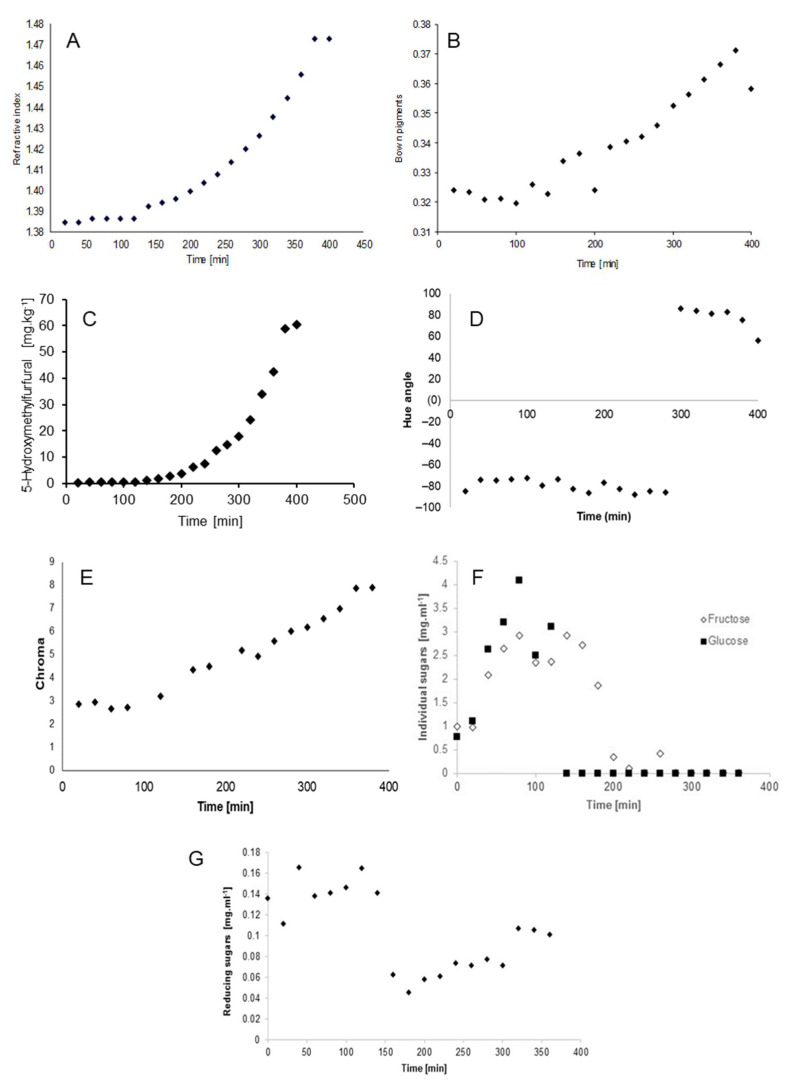
Effect of thermal-based processing on refractive index (**A**), brown pigments (**B**), 5-hydroxymethylfurfural (**C**), hue angle (**D**), chroma (**E**), individual sugars (**F**), and reducing sugars (**G**) during the *água-mel* production process. Statistical differences were assessed by repeated-measures ANOVA.

The refractive index (RI) measures the bending of a ray of light when passing from one medium into another, being an accurate indicator of the concentration of total soluble solids present in liquid samples. In this study, this parameter increased exponentially along the *água-mel* production process (from 1.38478 at time zero to 1.47285 after 400 min) (Figure 1A), probably due to the loss of moisture, in line with previous studies [1]. In particular, this 106% increase in the RI followed a first-order kinetics, with a constant rate of 0.1629 min^−1^ and r = 0.9008 (Table 1).

A similar behavior (k = 0.0004, r = 0.8791) was found for the evolution in brown pigments (BP) during the production process, as can be seen in Figure 1B. The content of BP increased as the heating time increased, which might be due to the occurrence of Maillard reactions as a result of the concentration of reacting species because of the decreased water content in *água-mel* [9,18]. In this respect, some researchers have reported that the brown color development in foods is particularly related to the content of reducing sugars [19,20]. A closer look at Figure 1B shows an initial induction period, which is followed by a steep increase in the formation of BP over time. This is in line with the results reported by Laroque et al., who investigated the evolution of Maillard reaction products from a shrimp hydrolysate with five different reducing sugars [21]. The authors reported that the induction period largely depends on the sugar used, being shorter for pentoses when compared to hexoses [21]. Kinetic modeling of our data demonstrated that BP changes during the production of *água-mel* adequately fit both zero- and first-order reaction equations (Table 1). The low production rate of BP that was observed in the samples of *água-mel* here analyzed is in accordance with the report by Ajandouz et al. about color development in different caramels [22]. Similarly, a first-order kinetics has also been used by Bostan et al. to describe the non-enzymic color development in glucose syrups stored at 45 °C and 55 °C [15,19,22,23]. On the other hand, Figure 1C represents the exponential increase in HMF along the 400 min heating of *água-mel*, from 0.21 mg kg^−1^ to 60.41 mg kg^−1^, at a constant rate of 0.0152 min^−1^ (r = 0.9855) (Table 1). HMF is one of the main products of carbohydrate degradation in foods by means of non-enzymatic browning reactions, whose content in saccharidic foodstuff has been demonstrated to greatly increase during thermal treatment [6]. The HMF evolution was also adequately described by a first-order reaction kinetic model (Table 1).

The results obtained for color parameters (i.e., H° and C*) are represented in Figure 1D–E. As for the hue angle (H°), it is observed that samples were initially located in the second quadrant from 0 to 300 min along the heating treatment, whereas H° values evolved to the first quadrant during the final 100 min of the processing, which corresponds to the dark red-orange zone, as previously reported in other studies [1]. The hue angle was described by a zero-order kinetic model, as can be seen in Table 1. On the other hand, the chroma (C*) parameter showed a gradual increase with processing time, from 2.850 to 7.890. Accordingly, our results indicate a considerable color increase of *água-mel* samples during processing, which could be attributed to the accumulation of BP. First-order kinetic models were adequate to describe the C* variation (Table 1), as previously reported [1].

Regarding sugars, glucose and fructose were the main species present in *água-mel*, whereas the concentration of sucrose was negligible. As shown in Figure 1F–G, the decrease in both individual and total reducing sugars after 150 min of thermal processing could be justified by carbohydrate degradation reactions, which was in turn accompanied by a steep increase in the formation of BP and HMF (Figure 1B,C), as discussed above. The degradation of sugars was represented by first-order kinetic models (Table 1) [23,24,25].

## 4. Conclusions

In this work, we investigated the physicochemical changes occurring along the thermal-based production of *água-mel* in terms of soluble solids, color parameters (hue angle, H°; chroma, C*), sugar content (total reducing sugars, glucose, fructose), and pigment content (total brown pigments, 5-hydroxymethylfurfural). Our results complement previous studies conducted on this traditional Portuguese honey-related food product, in which only a subset of the parameters here considered were analyzed. For instance, Figueira and Cavaco only investigated changes in viscosity, total soluble solids, and color parameters [1], whereas Miguel et al. focused on studying the evolution of the moisture, free acidity, and the content of 5-hydroxymethylfurfural, melanoidins, phenols, and fructose and glucose [2]. Therefore, this study provides complementary insights into the physicochemical evolution of *água-mel*, which is essential considering the extreme subjectivity of the production process. Interestingly, we observed a decrease in sugar contents after 150 min and the accumulation of brown pigments and 5-hydroxymethylfurfural, which together with the appearance of darker colors could be indicative of the occurrence of Maillard reactions. Furthermore, thermal processing also caused a significant increase in the refractive index, probably because of water losses. These results highlight the importance of controlling the entire production process (e.g., time, temperature) on the quality and related physicochemical properties of the final product.

## Figures and Tables

**Table 1:** Kinetic Models for The Physicochemical Parameters Evaluated along The *Água-mel* Production Process.

	Zero-Order Kinetic Model			First-Order Kinetic Model		
	K_0_ (min^−^^1^)	P_0_	r	K_1_ (min^−^^1^)	P_0_	r
Refractive index (RI)	1.655	0.8510	0.8975	0.1629	0.9517	0.9008
Brown pigments (BP)	0.0001	0.3115	0.8754	0.0004	0.3124	0.8791
5-hydroxymethylfurfural (HMF)	0.1525	−184.7	0.7321	0.0152	1.805	0.9855
Chroma (C*)	0.0149	1.895	0.9614	0.0032	2.409	0.9682
Hue angle (H°)	0.4784	−133.6	0.5765			
Reducing sugars (RS)	−0.0002	0.1394	0.3190	−0.0020	1.334	0.2486
Fructose	−0.0074	25.29	0.4923	0.0210	92.72	0.7027
Glucose	0.0200	12.80	0.5416	0.0111	11.14	0.6167

## Data Availability

The datasets used and/or analyzed during the current study are available from the corresponding author on reasonable request.

## Sample Availability

Samples of the compounds are not available from the authors.
